# Construction of an ER stress-related prognostic signature for predicting prognosis and screening the effective anti-tumor drug in osteosarcoma

**DOI:** 10.1186/s12967-023-04794-0

**Published:** 2024-01-16

**Authors:** Weidong Chen, Yan Liao, Pengxiao Sun, Jian Tu, Yutong Zou, Ji Fang, Ziyun Chen, Hongbo Li, Junkai Chen, Yuzhong Peng, Lili Wen, Xianbiao Xie

**Affiliations:** 1 Department of Musculoskeletal Oncology, The First Affiliated Hospital of Sun Yat-Sen University, Guangzhou, 510080 China; 2 Guangdong Provincial Key Laboratory of Orthopedics and Traumatology, The First Affiliated Hospital of Sun Yat-Sen University, Guangzhou, 510080 China; 3 State Key Laboratory of Organ Failure Research, Guangdong Provincial Key Laboratory of Renal Failure Research, Division of Nephrology, Nanfang Hospital, Southern Medical University, Guangzhou, 510515 China; 4 Macau University of Science and Technology, Macau, 999078 China; 5 Department of Anesthesiology, State Key Laboratory of Oncology in South China, Collaborative Innovation Center for Cancer Medicine, Sun Yat-Sen University Cancer Center, Guangzhou, 510060 China

**Keywords:** Osteosarcoma, ER stress, Prognostic signature, STC2, Tumor microenvironment, ISOX

## Abstract

**Background:**

Osteosarcoma is the most common malignant primary bone tumor in infants and adolescents. The lack of understanding of the molecular mechanisms underlying osteosarcoma progression and metastasis has contributed to a plateau in the development of current therapies. Endoplasmic reticulum (ER) stress has emerged as a significant contributor to the malignant progression of tumors, but its potential regulatory mechanisms in osteosarcoma progression remain unknown.

**Methods:**

In this study, we collected RNA sequencing and clinical data of osteosarcoma from The TCGA, GSE21257, and GSE33382 cohorts. Differentially expressed analysis and the least absolute shrinkage and selection operator regression analysis were conducted to identify prognostic genes and construct an ER stress-related prognostic signature (ERSRPS). Survival analysis and time dependent ROC analysis were performed to evaluate the predictive performance of the constructed prognostic signature. The “ESTIMATE” package and ssGSEA algorithm were utilized to evaluate the differences in immune cells infiltration between the groups. Cell-based assays, including CCK-8, colony formation, and transwell assays and co-culture system were performed to assess the effects of the target gene and small molecular drug in osteosarcoma. Animal models were employed to assess the anti-osteosarcoma effects of small molecular drug.

**Results:**

Five genes (BLC2, MAGEA3, MAP3K5, STC2, TXNDC12) were identified to construct an ERSRPS. The ER stress-related gene Stanniocalcin 2 (STC2) was identified as a risk gene in this signature. Additionally, STC2 knockdown significantly inhibited osteosarcoma cell proliferation, migration, and invasion. Furthermore, the ER stress-related gene STC2 was found to downregulate the expression of MHC-I molecules in osteosarcoma cells, and mediate immune responses through influencing the infiltration and modulating the function of CD8+ T cells. Patients categorized by risk scores showed distinct immune status, and immunotherapy response. ISOX was subsequently identified and validated as an effective anti-osteosarcoma drug through a combination of CMap database screening and in vitro and in vivo experiments.

**Conclusion:**

The ERSRPS may guide personalized treatment decisions for osteosarcoma, and ISOX holds promise for repurposing in osteosarcoma treatment.

**Supplementary Information:**

The online version contains supplementary material available at 10.1186/s12967-023-04794-0.

## Introduction

Osteosarcoma is the most common primary malignant bone tumor that predominantly affects infants and adolescents. Although wide-margin surgical resection combined with neoadjuvant chemotherapy has significantly improved the 5-year survival rate of patients to approximately 60%, the emergence of lung metastases and local recurrence worsen the prognosis, reducing the survival rate to less than 20% [1, 2]. Treatment of osteosarcoma has stagnated without significant therapeutic efficacy advances over the past decades. The genetic heterogeneity of osteosarcoma may further complicate treatment, as patients with identical clinical and pathological characteristics respond differently to the same treatment. This highlights the inadequacy of current therapeutic strategies and the urgent need to investigate novel therapeutic approaches. Therefore, a more comprehensive understanding of the complex interplay between the novel molecules and the malignant progression of osteosarcoma is necessary to identify potential therapeutic targets and improve patient outcomes.

According to many studies, persistent active ER stress has emerged as a hallmark of cancer, regulating multiple pro-tumor characteristics in cancer cells and orchestrating diverse immunomodulatory mechanisms that promote malignant progression by dynamically reprogramming the function of innate and adaptive immune cells [3, 4]. Additionally, it is believed that aberrant activation of ER stress regulates treatment response to chemotherapy, targeted therapies, and immunotherapy [4]. This stress is caused by the disruption of protein folding homeostasis by multiple factors, such as hypoxia, low pH, metabolic stress, and oncogenic abnormalities in the tumor microenvironment (TME) [5–7]. These conditions alter the protein folding capacity of the ER in cancer and immune cells, leading to the accumulation of harmful misfolded/unfolded proteins and ER stress. In response, the unfolded protein response (UPR) is activated to restore ER homeostasis and adapt to tumor development mainly through three pathways, including inositol-requiring enzyme 1α (IRE1α), protein kinase RNA-like endoplasmic reticulum kinase (PERK), and activating transcription factor 6 (ATF6) [8]. Research showed that activation of UPR may lead to the suppression of surface expression of MHC class I (MHC-I) molecules, which is mediated by the upregulation of X-box binding protein 1s(XBP1s) and ATF6, ultimately resulting in immune evasion by tumor cells [9]. The activation of the IRE1α-XBP1 pathway promotes the expression of tissue protease, programmed death-ligand 1 (PD-L1), and arginase 1(Arg1) in macrophages [10]. These factors contribute to the inhibition of T cell activation and proliferation, as well as the promotion of extracellular matrix remodeling, ultimately facilitating tumor cell invasion and metastasis. Furthermore, recent studies had showed that myeloid-derived suppressor cells (MDSCs) regulate antitumor immunity through the PERK pathway by expressing T cell inhibitory factors and nuclear factor erythroid 2-related factor 2 (NRF2). These enable MDSCs to suppress T cell activation and proliferation, promote T cell apoptosis, and enhance immunosuppressive cytokine production [11]. According to these studies, targeting ER stress may be a promising therapeutic approach for the treatment of malignancies. Therefore, a comprehensively analysis of endoplasmic reticulum stress-related genes (ERSRGs) and further exploration of their biological characteristics might be helpful in identifying novel prognostic biomarkers and developing effective therapeutic strategies for osteosarcoma.

In this study, we developed a novel ER stress-related prognostic signature (ERSRPS) for osteosarcoma based on five differentially-expressed ERSRGs; among them, Stanniocalcin 2 (STC2) was determined to be the risk gene associated with an unfavorable prognosis in ER stress signaling. The ER stress related gene STC2 was found to be highly expressed in osteosarcoma, and STC2 knockdown significantly inhibited the proliferation and migration of osteosarcoma cells. STC2 knockdown also resulted in the upregulation of MHC-I molecule expression in osteosarcoma cells and the restoration of CD8+ T cells activation. Additionally, our risk model revealed a close association between ER stress and immune cell infiltration, and immune checkpoint blockades (ICBs) response. Furthermore, the CMap database was utilized to screen ISOX as a potential small molecule drug for high-risk patients and validated its anti-osteosarcoma efficacy.

## Materials and methods

### Dataset source and preprocessing

Gene Expression Omnibus (GEO) databases GSE21257 (n = 53) and GSE33382 (n = 87) were downloaded (https://www.ncbi.nlm.nih.gov/), and the Therapeutically Applicable Research to Generate Effective Treatment-Osteosarcoma (TARGET-OS) dataset (n = 85) was obtained from the TARGET database (https://portal.gdc.cancer.gov/). TARGET-OS and GSE 21257 both contain RNA sequences and clinical information, whereas GSE33382 lacks corresponding clinical information. We obtained a set of 258 ER stress-related genes (GOBP_RESPONSE_TO_ENDOPLASMIC_RETICULUM_STRESS) from the MSigDB database (MSigDB; https://www.gsea-msigdb.org/gsea/msigdb/index.jsp) [12].

### Unsupervised clustering of ERSRGs and functional enrichment analysis

Unsupervised clustering methods were employed to classify patients into discrete molecular subtypes based on the ERSRGs expression derived from the TARGET-OS cohort. The number of clusters and their stability were determined using an R package named **“**ConsensusClusterPlus” [13]. The partitioning around medoids clustering algorithm was executed with 1,000 initial resampling and 50 iterations. The consensus matrix, cumulative distribution function (CDF), and relative change in area under the CDF curve were utilized to determine the optimal number of clusters. Kaplan–Meier survival analysis was generated to evaluate the overall survival rates of each subtype. Additionally, Gene Ontology (GO) annotation was utilized to analyze the functional characteristics associated with the various clusters based on comprehensive gene expression profiles.

### Construction and validation of the endoplasmic reticulum stress-related prognostic signature

Firstly, the differentially expressed ERSRGs were analyzed using the “limma” package to identify genes associated with osteosarcoma using TARGET-OS data. Significant variables (*p* < 0.05) were then subjected to least absolute shrinkage and selection operator (LASSO) analysis using the “glmnet” package [14]. The candidate genes were then identified based on the optimal penalty parameter λ determined by the 1-SE (standard error) criterion. Finally, the differentially expressed prognostic ERSRGs were utilized to construct an optimal ERSRPS. Risk scores were computed by aggregating the expression and corresponding coefficient of each prognostic ERSRGs. Patients were then classified as low- or high-risk based on their median risk scores. The prognostic performance of the ERSRPS was assessed using Kaplan–Meier analysis and time-dependent ROC analysis. The degree of generalization of the ERSRPS was confirmed by using data from the external independent validation cohorts.

A nomogram, integrating the risk scores with clinical parameters (age, gender, metastasis status), was constructed using Cox regression coefficients to predict 1-year, 3-year, and 5-year overall survival in osteosarcoma patients. The R packages “rms”, “regplot” and “Hmisc” were utilized in this process. We then used the calibration and ROC curve to estimate the consistency between predicted survival and actual survival.

### Evaluation of the immunogenomic landscape

The tumor microenvironment (TME) is mainly composed of stromal cells and immune cells. We used the ESTIMATE algorithm to calculate the immune score, stromal score, estimate score, and tumor purity all samples. Additionally, the abundance of 29 immune cells and immune-related molecules was evaluated for each osteosarcoma sample in TARGET-OS using the ssGSEA algorithm. Furthermore, we acquired two pretreatment tumor expression profiles (GSE35640 and GSE78220) from cohorts involving immune checkpoint blockade (ICB) treatment to evaluate the likely response of different subgroups to immunotherapy [15, 16].

### Cell culture

Cell lines (hFOB1.19, U2OS, U2R, HOS, MNNG/HOS, 143B, SJSA-1, G292, SAOS2, MG63, and ZOS) were obtained from the First Affiliated Hospital of Sun Yat-sen University. The cell line hFOB 1.19 was cultured in DMED/F12k medium supplemented with 10% fetal bovine serum, while the other cell lines were cultured in high-glucose DMED medium supplemented with 10% fetal bovine serum. The cells were maintained in an incubator at 37 °C and 5% CO_2_. The absence of mycoplasma contamination in all cell lines was confirmed on a regular basis through testing.

### Lentiviral transduction for stable cell lines

The lentiviruses packaging STC2 shRNA (targeting sequences: #sh1: GGTGAGCGAGGTAGCAAGA, #sh2: CACAGGTTCGGCTGCATAA) were purchased from Tsingke Biotechnology (Beijing, China). These sequences were cloned into the Plko.1-puro vector individually. Lentiviral packaging experiments were conducted using Lipofectamine 3000 (Invitrogen), as manufacture described. STC2 knockdown plasmid was co-transfected with the packaging plasmids (pMD2.G, psPAX2) into HEK293T cells. The lentivirus was collected 48 h after transfection. To generate stable STC2 knockdown cell lines, 143B and SJSA-1 osteosarcoma cells were transduced with lentiviruses and then selected with puromycin (2 µg/ml, Biosharp) after 2 weeks of production.

### RNA extraction and quantitative real-time PCR (RT-qPCR) analysis

The TRIzol reagent (Invitrogen) was used to extract RNA from samples. Total RNA was reverse-transcribed into cDNA using the PrimeScript RT reagent kit (Takara). Real time RT-PCR was performed using qRT-PCR SYBR Green Kit (Takara) with 7500 Fast Real-Time PCR System (Applied Biosystem). GAPDH expression was used as an internal reference. The following primers were used: STC2-RE, 5ʹ-TTTCCAGCGTTGTGCAGAAAA-3ʹ and 5ʹ-GGGTGTGGCGTGTTTGAATG-3ʹ; HLA-A-RE, 5ʹ-GCTGTGAGGGACACATCAGAG-3ʹ and 5ʹ-AAAAGGAGGGAGTTACACTCAGG-3ʹ; HLA-B-RE, 5ʹ-CAGCCGTACATGCTCTGGA-3ʹ and 5ʹ-CAGTTCGTGAGGTTCGACAG-3ʹ; B2M-RE, 5ʹ-CGGCAGGCATACTCATCTTTT-3ʹ and 5ʹ-GAGGCTATCCAGCGTACTCCA-3ʹ; GAPDH, 5ʹ-GGCTGTTGTCATACTTCTCATGG-3ʹ and 5ʹ-GGAGCGAGATCCCTCCAAAAT-3ʹ.

### Western blotting

Total cellular and tissue proteins were extracted with RIPA lysis buffer (Biosharp) supplemented with protease and phosphatase inhibitor. After thorough lysis, the samples were centrifuged at 12,000*g* for 10 min to remove cell or tissue debris. The protein samples were denatured for 10 min at 100 °C and separated by SDS-PAGE. Subsequently, the separated proteins were transferred onto 0.45 µm PVDF membranes for 90 min at 4 °C and 100 V. The membranes were blocked in 5% BSA with TBST for 1 h at room temperature before being incubated overnight at 4 °C with the primary antibody. After washing three times with TBST, the membranes were incubated with the corresponding secondary antibodies. The immunoreactive proteins were visualized using ECL detection reagents. The primary antibodies used for western blotting assay were: anti-STC2 (1:200, 10314-1-AP, Proteintech), anti-HLA-ABC (1:4000, 15240-1-AP, Proteintech), anti-B2M (1:4000, 13511-1-AP, Proteintech), GAPDH (1:5000, 10494-1-AP, Proteintech).

### Cell proliferation

Cell proliferation activity was evaluated by the CCK-8 assay. Briefly, osteosarcoma cells were seeded in 96-well plates. After 48 h of antitumor drugs treatment, 10 µl CCK-8 solution was added to the cells, and the absorbance was measured at 450 nm following a 3-h incubation period. Antitumor drugs screened from the Connectivity Map database, including Calyculin, Deforolimus, WYE-354, and Pyroxamide, were purchased from TargetMol (Wellesley Hills, MA, USA) and ISOX was purchased from MedChemExpress (Shanghai, China).

### Colony formation assay

Logarithmic phase osteosarcoma cells were digested, centrifuged, and resuspended. Then, 500 cells were seeded in 2 ml of culture medium in each well of a 6-well plate. After 2 weeks of incubation, the cells were fixed with 4% paraformaldehyde, stained with crystal violet, and photographed. The number of clones was calculated using ImageJ software.

### Migration and invasion assays

Migration and invasion assays were conducted utilizing a 24-well Transwell system with polycarbonate filters (8 µm pores, Corning) with or without a precoating of extracellular matrix (BD Biosciences). Briefly, 200 µl of a suspension containing 50,000 osteosarcoma cells in serum-free DMEM was added to the upper chamber of the 24-well plate, and 500 µl of DMEM containing 10% FBS was added to the lower chamber. After 10 h incubation at 37 °C, the cells in the Transwell system were fixed with 4% paraformaldehyde for 15 min and stained with 0.25% crystal violet for 30 min. The cells that migrated to the lower chamber were photographed and counted.

### Isolation and activation of peripheral blood mononuclear cells

Peripheral blood mononuclear cells (PBMCs) of healthy donors (n = 3) were isolated by Ficoll-Hypaque density gradient centrifugation and then cultured in RPMI-1640 medium supplemented with 10% FBS. In addition, the PBMCs were activated by adding 25 µl/mL of Human CD3/CD28 T Cell Activator (Cat.10991, STEMCELL Technologies) in combination of 1.5 ng/ml IL-2 (Cat.791902, BioLegend) for 72 h at 37 °C.

### Co-cultures system

The pre-activated T cells were co-cultured with 143B and SJSA-1 osteosarcoma cells by using a transwell culture system with 0.4 μm pore polyester membrane. Briefly, the pre-activated T cells were seeded in 6-well plates at 1 × 10^6^ cells/well, and osteosarcoma cells were seeded at 1 × 10^5^ cells/well in Transwell chambers. After 48 h of co-culture, the suspensions CD8+ T cells were harvested and resuspended in staining buffer (DPBS containing 3% FBS) with the indicated antibodies: APC anti-human CD8 (Cat.344721, BioLegend), PE anti-human/mouse Granzyme B (Cat.372207, BioLegend), Brilliant Violet 605™ anti-human IFN-γ (Cat.502535, BioLegend), and analyzed by flow cytometry.

### Flow cytometry

Tumors were enzymatically digested to single cell suspensions and filtered twice through 70 μm filters. Cells were then resuspended in staining buffer (DPBS containing 3% FBS) and stained with the indicated antibodies: APC/Cyanine7 anti-mouse CD45 (Cat. 147717, BioLegend), PE anti-mouse CD3 (Cat. 100205, BioLegend), APC anti-mouse CD8a (Cat. 100711, BioLegend), PerCP/Cyanine5.5 anti-human/mouse Granzyme B (Cat. 372211, BioLegend), VioletFluor™ 450 Anti-Mouse IFN gamma (Cat 75-7311-U025, Tonbo Bioscience), Brilliant Violet 510™ anti-mouse/human CD11b (Cat. 101245, BioLegend), PE anti-mouse Ly-6C (Cat. 128007, BioLegend), FITC anti-mouse Ly-6G (Cat.127605, BioLegend), PerCP/Cyanine5.5 anti-mouse F4/80 (Cat. 123127, BioLegend). Flow cytometry was performed on BD FACS Symphony machines. Flow analyses were performed using FlowJo 10.6.1 software (TreeStar). All flow antibodies were used at a 1:200 dilution.

### Immunohistochemistry (IHC)

Osteosarcoma specimen tissue sections were initially deparaffinized prior to antigen retrieval with an EDTA Antigen Retrieval Solution. Subsequently, the sections were treated with 3% H_2_O_2_ and blocked with 5% goat serum. The sections were then incubated overnight at 4 °C with primary antibodies, including anti-STC2 (1:200, Cat.10314-1-AP, Proteintech), anti-HLA-ABC (1:4000, Cat.15240-1-AP, Proteintech), anti-B2M (1:4000, Cat.13511-1-AP, Proteintech), and CD8α antibody (1:400, Cat.85336S, Cell Signaling Technology). The IHC staining was performed using Anti-Rabbit/Mouse HRP Conjugate, and the detection was carried out using 3,3ʹ-diaminobenzidine (DAB) (DAKO). Finally, the sections were counterstained with hematoxylin. Three random images were obtained for each tumor sample using a microscope. The levels of IHC staining were quantified according to immunoreactive score systems and the cut-off value for defining high or low STC2 expression in IHC was the median IHC score.

### Animal models

All animal experiments were approved by the Animal Research Committee of The First Affiliated Hospital of Sun Yat-sen University and conducted in accordance with the established guidelines for the Use and Care of Laboratory Animals. Briefly, 1 × 10^6^ wild-type SJSA-1 cells were suspended in 30 µl PBS and injected via a 30-gauge needle into the proximal tibia through the cortex of the anterior tuberosity in male BALB/C nude mice (4 weeks old). In addition, 2 × 10^6^ wild-type K7M2 osteosarcoma cells were subcutaneously injected into syngeneic BALB/C mice to evaluate the potential influence of the immune system in response to the anti-tumor drug treatment. The mice received intraperitoneally (i.p.) administered of vehicle control (2% DMSO/30% PEG 300/ddH2O) or ISOX at doses of 50 or 100 mg/kg daily. The mice were monitored every 3 days for 3 weeks. Five mice from each group were sacrificed at random when the tumors attained an approximately spherical shape. The subcutaneous and xenograft tumors were harvested, photographed, weighed. Tumor size was calculated using the formula: width^2^ × length × π/6, as described previously [17]. The number of metastatic nodules in the lungs was counted. The investigators conducting the experiment and assessing the outcomes were blinded to the group allocation.

### Statistical analysis

All statistical analyses and graph visualization were conducted using R v4.2.1 (http://www.r-project.org) or GraphPad Prism (version 9.0). All experiments were performed in triplicate, and the representative data from one experiment are presented. Statistical analyses were conducted using Student’s t-test or Chi-square test. Overall survival was analyzed using the Kaplan–Meier method and compared using the log-rank test. Differences with *p*-values < 0.05 were considered statistically significant.

## Results

### Identification of ER stress related-clusters and related biological processes in osteosarcoma

**Fig. 1:**
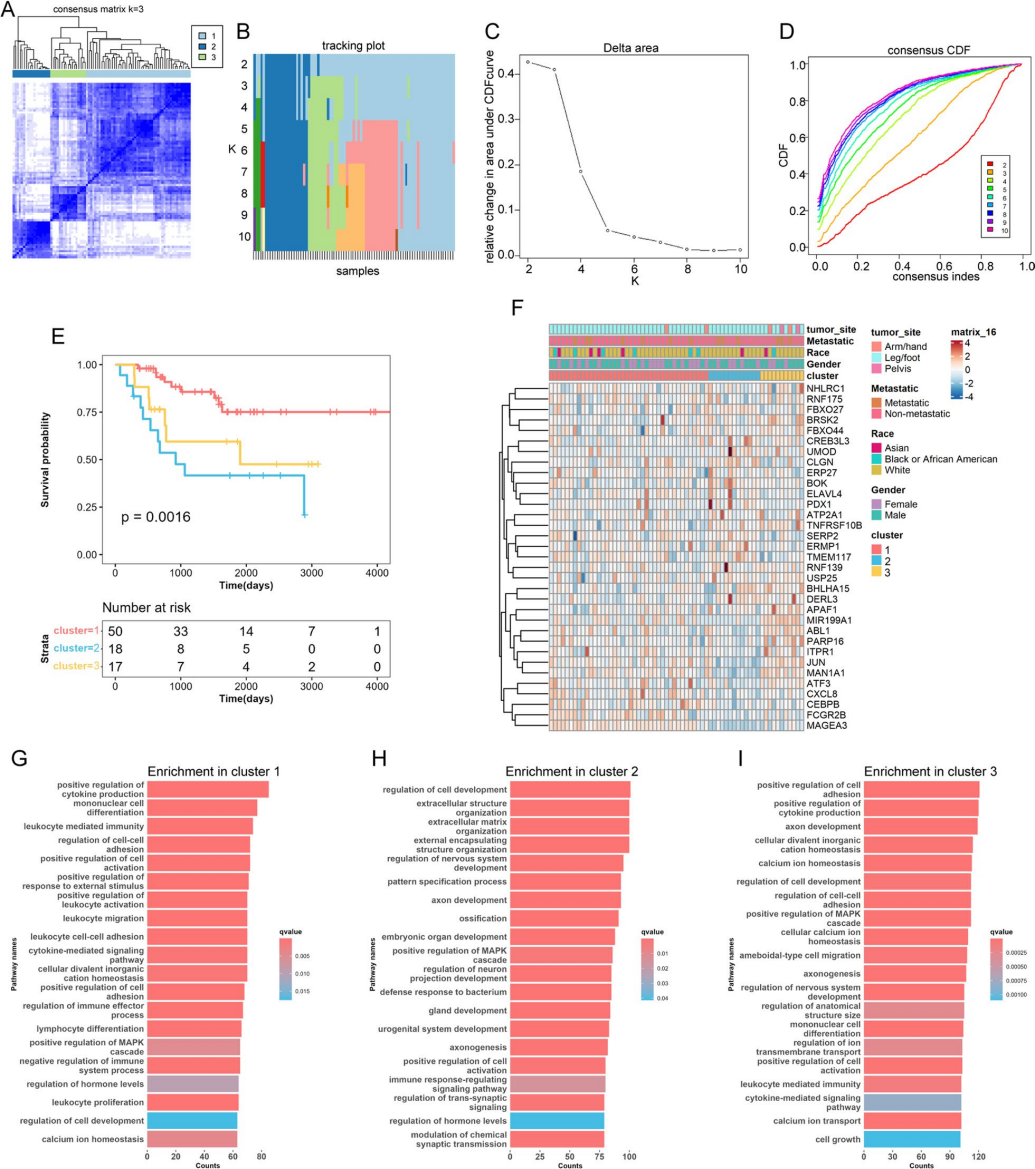
Clusters of ER stress-related genes (ERSRGs) and biological characteristics in osteosarcoma. **A** Consensus matrices of the TARGET-OS cohort for k = 3; **B** the sample distribution changed with k valued 2 to 10; **C** the relative change in area under the CDF curve; **D** consensus clustering cumulative distribution function (CDF) with k valued 2 to 10. **E** Survival analysis of the three ERSRG clusters. **F** Unsupervised clustering of the top 33 ERSRGs. **G**–**I** Gene Ontology (GO) annotation of the three ERSRG clusters

We performed consensus clustering analysis based on 258 ER stress-related genes within the osteosarcoma cases in the TARGET-OS database, aiming to explore the relationship between ER stress and osteosarcoma subgroups. The analysis identified three distinct clusters (k = 3) based on the least amount of crossover in the consensus matrix (Fig. 1A, B), no significant shift in the area under the curve (Fig. 1C), and the uniform trend in the CDF (Fig. 1D). Accordingly, the entire cohort was stratified into three distinct clusters, and survival analysis demonstrated that cluster 2 patients suffered the worst prognosis, while cluster 1 patients had the best prognosis (Fig. 1E). We then generated a heatmap to illustrate the relationship between ER stress related genes and various clinical characteristics, such as gender, age, metastasis status, and primary tumor site. No substantial differences were detected in clinical characteristics across the three clusters, indicating a similar distribution similarity in these aspects (Fig. 1F). Gene ontology (GO) analysis indicated that the differentially expressed genes (DEGs) in cluster 1 were mainly enriched in biological processes associated with immune cell infiltration and their activation, whereas in cluster 2, they were primarily associated with extracellular matrix organization (Fig. 1G–I). These findings suggested that ER stress is associated with the survival of osteosarcoma patients and may be involve in immune cells infiltration.

### Construction of a prognostic risk score model for osteosarcoma based on ERSRGs

**Fig. 2:**
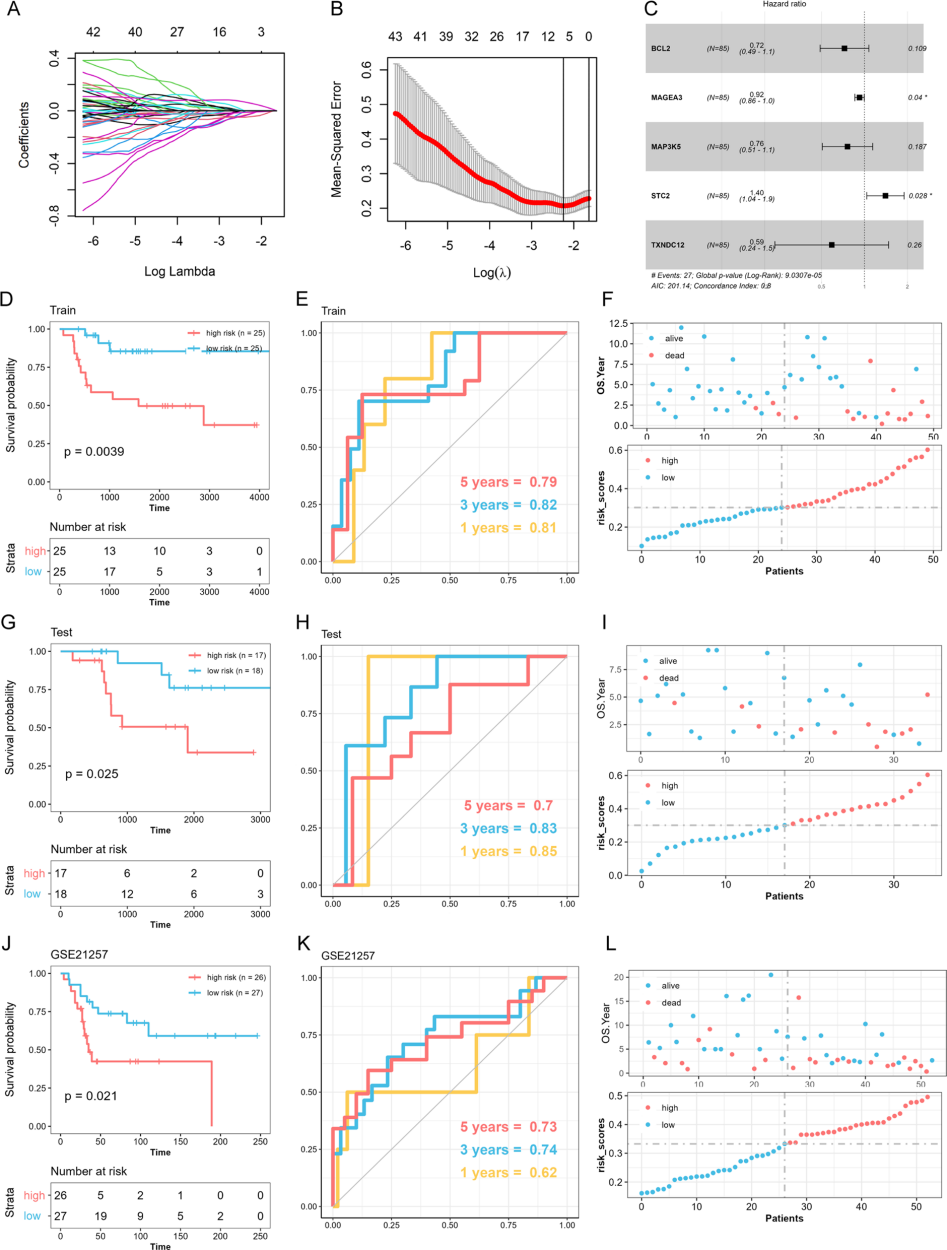
Construction of an ER Stress-related prognostic signature (ERSRPS). **A** Coefficient profiles of the differentially expressed related genes and **B** Identification of the best parameter (lambda) in the LASSO. **C** Five genes retained to construct the optimal ERSRPS. **D**, **G**, **J** Kaplan–Meier analysis of overall survival. **E**, **H**, **K** Receiver operating characteristic (ROC) analysis of the ERSRPS in predicting the 1-, 3-, and 5-year overall survival. **F**, **I**, **L** Distribution plots of the risk score and overall survival status in the training, testing groups and GSE21257 cohort

To further elucidate the impact of ERSRGs on the progression of osteosarcoma, we initially explored the mRNA expression profiles of the selected ERSRGs within the GSE33382 cohort. We identified DEGs using the screening criterion *p* < 0.05 by comparing the expression levels of tumor and normal samples. We then used the “rsample package” in R to randomly classify the TARGET-OS patients into training (n = 50) and testing (n = 35) groups with a ratio of 6:4. Furthermore, we utilized LASSO penalized Cox regression analysis to identify DEGs and prognostic ERSRGs (Fig. 2A, B). The penalty parameter was selected based on the minimum criterion. Based on this analysis, five prognostic ERSRGs (BLC2, MAGEA3, MAP3K5, STC2, and TXNDC12) were identified and used to construct a prognostic risk score model (Fig. 2C). The risk score calculation was performed using the following formula: risk score = (− 0.020* BLC2) + (− 0.010* MAGEA3) + (− 0.043* MAP3K5) + (0.057* STC2) + (− 0.022* TXNDC12).

In the training group, the median risk score was used to classify 25 patients as high-risk and 25 as low-risk. The Kaplan–Meier curve indicated higher mortality rates and shorter overall survival times in the high-risk group (Fig. 2D). The prognostic model demonstrated high sensitivity and specificity, with an area under the ROC curve (AUC) of 0.81 for 1-year, 0.82 for 3-year, and 0.79 for 5-year survival predictions (Fig. 2E). The risk score plot and living status revealed that the high-risk group had lower survival status and a shorter survival time (Fig. 2F). Additionally, the independent testing group and GSE21257 cohort were used as validation groups. Similar findings were observed using the same formula and median risk score (Fig. 2G–L).

### Development of a clinical nomogram and pan-cancer analysis

**Fig. 3:**
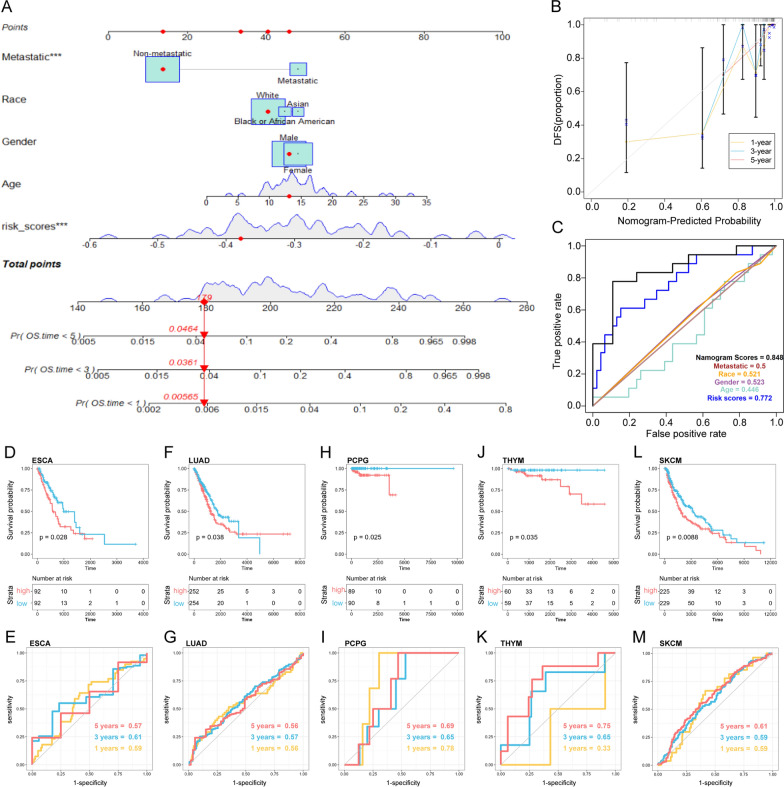
Development of a clinical nomogram and pan-cancer analysis. **A** Nomogram based on the ERSRPS and metastasis. **B** Calibration curves for internal validation of the nomogram. **C** Receiver operating characteristic (ROC) curves of the nomogram in predicting the overall survival. Kaplan–Meier and ROC analysis of ESCA (**D**, **E**), LUAD (**F**, **G**), PCPG (**H**, **I**), THYM (**J**, **K**), SKCM (**L**, **M**)

Subsequently, we constructed a nomogram for predicting overall survival in the TARGET-OS cohort, incorporating both clinical parameters and the ERSRGs-based risk scores (Fig. 3A). The calibration plot for the internal validation of the nomogram demonstrated consistency between the nomogram-predicted probability and actual observations (Fig. 3B). The combined nomogram exhibited an area under the curve (AUC) of 0.848, increasing the efficiency of the other clinical factors for predicting overall survival (Fig. 3C). Furthermore, the risk model exhibited prognostic power for overall survival in several cancer types among the 33 pan-cancer types, including ESCA (*p* = 0.028) (Fig. 3D, E), LUAD (*p* = 0.038) (Fig. 3F, G), PCPG (*p* = 0.025) (Fig. 3H, I), THYM (*p* = 0.035) (Fig. 3J, K), and SKCM (*p* = 0.0088) (Fig. 3L, M).

### ER stress related gene—STC2 promotes osteosarcoma growth and metastasis

**Fig. 4:**
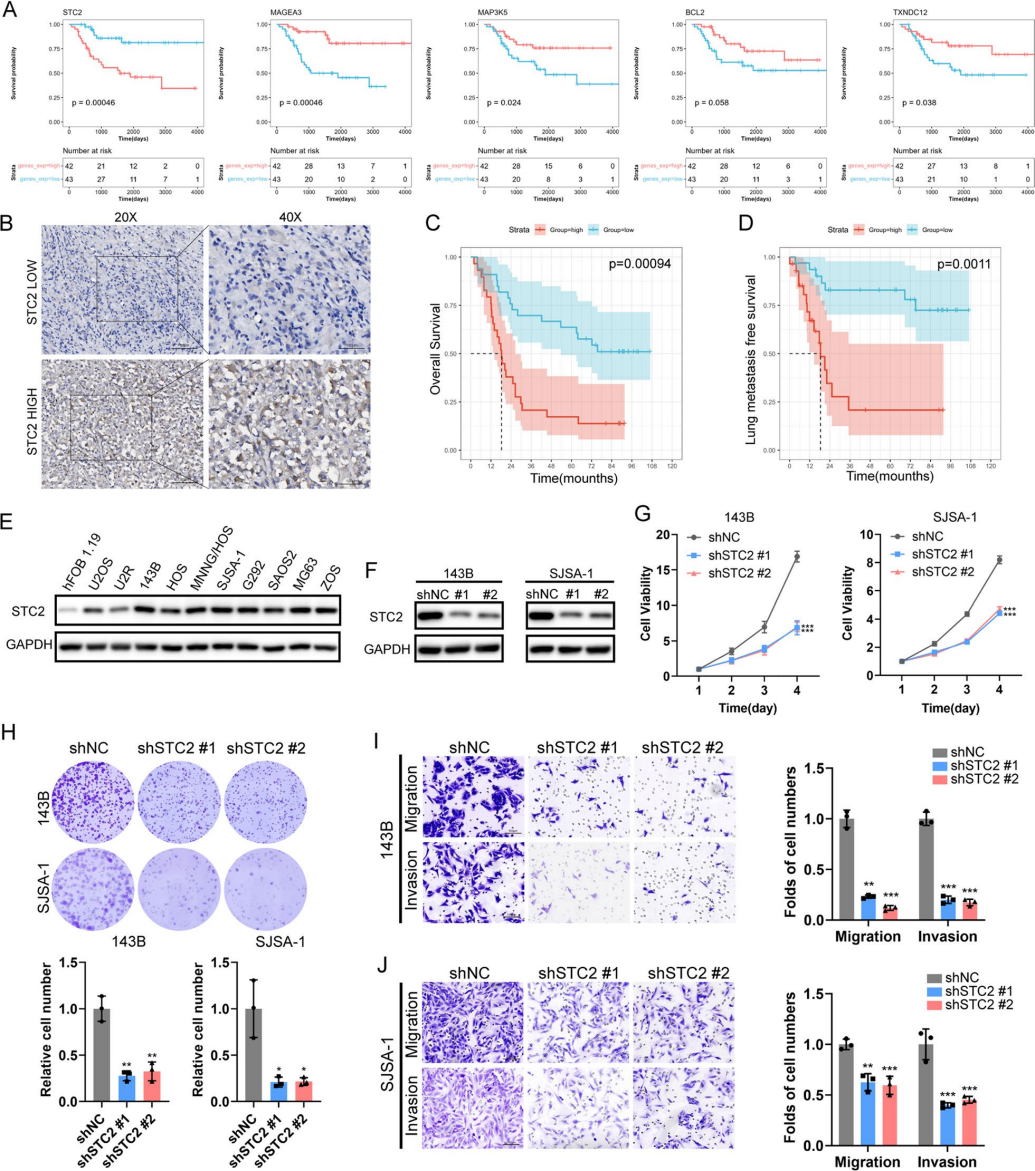
STC2 correlates with poor prognosis and promotes osteosarcoma proliferation and metastasis in vitro. **A** Kaplan–Meier survival analysis of STC2 in TARGET-OS cohort. **B** IHC staining of STC2 in 62 paraffin-embedded osteosarcoma tissues. Scale bar: 50 μm (left) and 100 μm (right). **C**, **D** Kaplan–Meier survival analysis of osteosarcoma patients stratified by STC2 expression level (n = 62). **E** Protein levels of STC2 in HFOB1.19 cells and several osteosarcoma cell lines by Western blot analysis. **F** Knockdown of STC2 in 143B and SJSA-1 osteosarcoma cells. **G** Cell proliferation, **H** colony formation, **I**, **J** Migration and invasion ability of STC2 knockdown 143B and SJSA-1 cells. *p < 0.05, **p < 0.01, ***p < 0.001

Kaplan–Meier curve analysis demonstrated that high expression of the four prognostic ERSRGs, including BCL2, MAGEA3, MAP3K5, and TXNDC12, were significantly associated with a favorable overall survival. In contrast, high expression of the ER stress related gene STC2 was associated with an unfavorable overall survival in this patient population (Fig. 4A). To further investigate the clinical significance of STC2 in osteosarcoma, IHC staining was conducted to evaluate STC2 expression in our clinical osteosarcoma specimens (n = 62) (Fig. 4B, Additional file 1: Table S1). The results demonstrated that patients with high STC2 expression had lower 5-year overall survival and lung metastasis-free survival rates than those with low expression (Fig. 4C, D). Additionally, western blotting revealed that STC2 was up-regulated in osteosarcoma cells (Fig. 4E). To further elucidate the functional implications of STC2 in the malignant progression of osteosarcoma, two independent short hairpin RNA (shRNA) sequences were used to develop stable knockdown models of STC2 in SJSA-1 and 143B cells (Fig. 4F). Our results demonstrated that knockdown of STC2 exerted a substantial inhibitory effect on cell proliferation (Fig. 4G), colony formation (Fig. 4H) migration and invasion abilities (Fig. 4I, J) both in SJSA-1 and 143B cells.

### *ER stress related gene—STC2 influences CD8*+ *T cell infiltration and impairs their function*

**Fig. 5:**
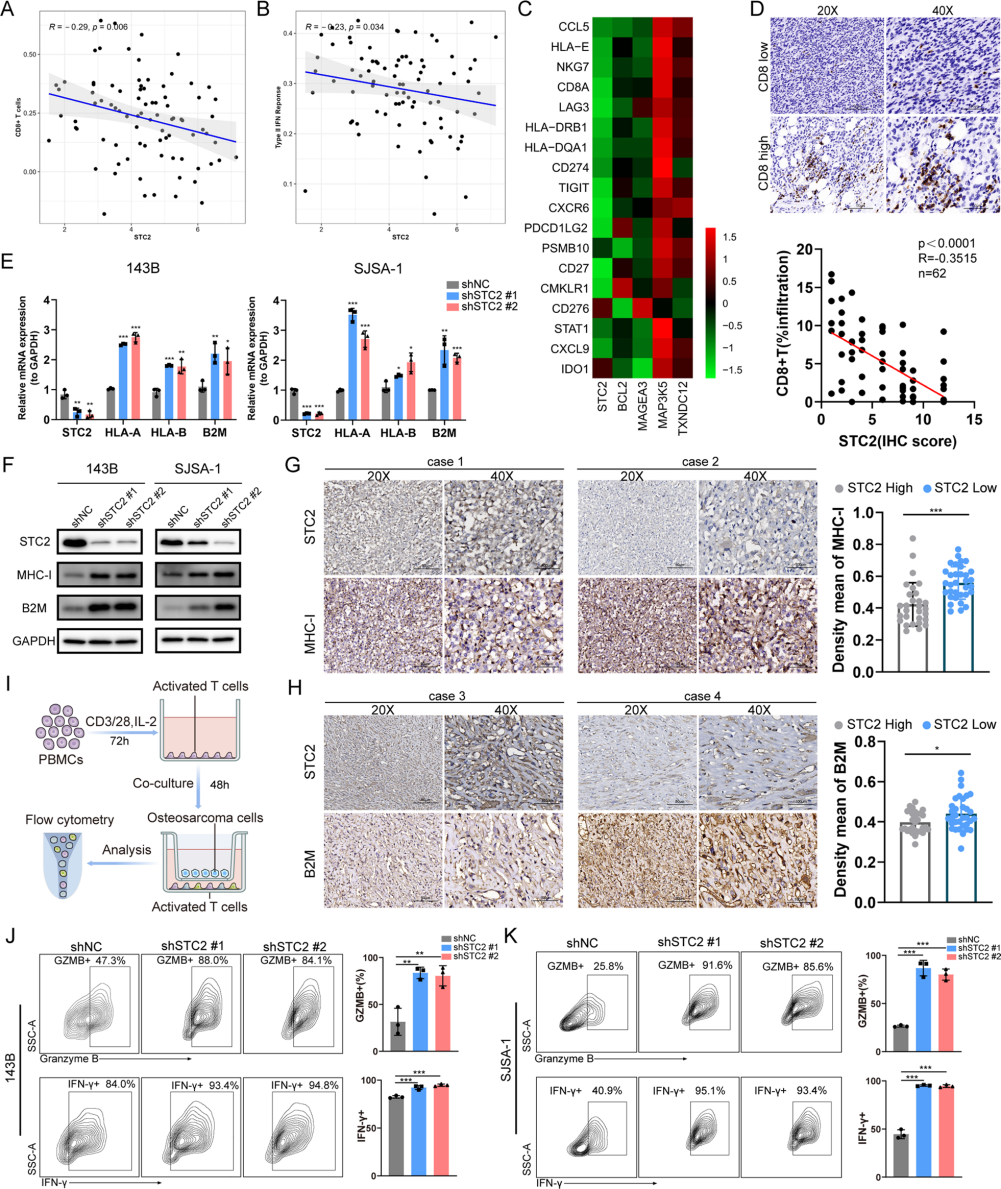
STC2 is associated with an immunosuppressive microenvironment in osteosarcoma patients. **A**, **B** Correlation analysis between STC2 and the infiltration of CD8+ T cells as well as the type II interferon response pathway in TARGET-OS cohort. **C** Correlation in mRNA expression between STC2 and type II interferon related genes in TARGET-OS cohort. **D** Correlation between protein levels of STC2 and CD8+ T cells infiltration was evaluated through immunohistochemistry staining in osteosarcoma patients (p < 0.0001, R =  − 0.3515, n = 62). **E**, **F** qPCR and Western blot analysis of the expression of MHC-I molecules in STC2 knockdown 143B and SJSA-1 cells. **G**, **H** Correlation between protein levels of STC2 and MHC-I molecules through immunohistochemistry staining in osteosarcoma patients. **I** Schematic workflow for osteosarcoma cell and T cells co-culture system. Naive T cells were in vitro stimulated with CD3/CD28 antibodies in the presence of interleukin (IL)-2 (1.5 ng/ml). **J**, **K** Activated T cells were co-cultured with osteosarcoma cells, and CD8+ T cells function markers were determined by flow cytometry. *p < 0.05, **p < 0.01, ***p < 0.001

To further elucidate the potential role of STC2 within the immune microenvironment, we performed a comprehensive correlation analysis between STC2 and the reported 29 immune-related gene sets, comprising a variety of immune cell types and functions [18]. Intriguingly, our findings demonstrated that STC2 exhibited a negative correlation with CD8+ T cell infiltration (R = − 0.29, *p* = 0.006) (Fig. 5A) and type II interferon response (R = − 0.23, *p* = 0.034) (Fig. 5B) in TARGET-OS cohort. Moreover, our results revealed a negative correlation between STC2 expression and a type II IFN-related gene signature comprising of 18 genes (Fig. 5C). In accordance with the TARGET-OS cohort, IHC staining from our osteosarcoma cohort revealed a correlation between protein expression level of STC2 and infiltration of CD8+ T cells (*p* < 0.0001, R =  − 0.3515, n = 62) (Fig. 5D, Additional file 2: Table S2). Previous studies revealed that the downregulation of MHC-I expression in cancer cells is a crucial factor that inhibits both the antitumor effect of type II IFN signaling and the infiltration of CD8+ T cells [19, 20]. Our results demonstrated a substantial upregulation of both RNA and protein levels of classical MHC-I molecules (HLA-A, HLA-B, and B2M) on STC2 knockdown (Fig. 5E, F). Additionally, IHC staining revealed a negative correlation between STC2 expression and the expression of MHC-I molecules in our osteosarcoma cohort (Fig. 5G, H, Additional file 2: Table S2). These findings implied that STC2 might mediate immune evasion in osteosarcoma by downregulating the expression of MHC-I molecules on the surface of tumor cells, thereby suppressing type II interferon response and reducing CD8+ T cells infiltration. Given that STC2 is a secreted glycoprotein, we then sought to explore its potential impact on modulating CD8+ T cell function. We conducted a co-culture system involving osteosarcoma cells and pre-activated T cells (Fig. 5I). Flow cytometry analysis suggested that CD8+ T cell exhibited higher expression levels of IFN-γ and granzyme B when co-cultured with STC2 knockdown osteosarcoma cells compared to those co-cultured with wild-type osteosarcoma cells (Fig. 5J, K). Together, our results suggested that ER stress related gene STC2 involved in immune regulation through reducing CD8+ T cell infiltration and impairing their function.

### Pan-cancer analysis of STC2

To acquire a more comprehensive understanding of the ER stress related gene STC2 in cancer development and immunity, we performed a pan-cancer analysis to investigate the aberrant expression of STC2 across 33 different cancer types in the TCGA-Pan-cancer cohort. The analysis revealed higher STC2 expression in 18 tumor types (Additional file 3: Figure S1A). The TIMER2.0 database revealed correlations between STC2 expression and various immune infiltrating cells (Additional file 3: Figure S1B). Moreover, we assessed the correlation between STC2 expression and two established immunotherapy predictive biomarkers: tumor mutation burden (TMB) and microsatellite instability (MSI) [21, 22]. The findings indicated a positive correlation between STC2 expression and tumor mutation burden (TMB) in GBM, HNSC, KIRC, LAML, PRAD, READ, THYM, and UCEC (Additional file 3: Figure S1C). Furthermore, a positive correlation between STC2 expression and microsatellite instability (MSI) was observed in HNSC, LUAD, LUSC, THYM, and ACC (Additional file 3: Figure S1D). Spearman correlation analysis was then conducted to explore the associations between STC2 expression and immune checkpoint genes, including both inhibitory and stimulatory factors in pan-cancer analysis (Additional file 3: Figure S1E). We observed a significant positive correlation between STC2 and a wide range of immune checkpoint genes across various cancer types. These findings signified the involvement of the ER stress-related gene STC2 in the regulation of the tumor immune microenvironment across a variety of cancer types.

### The immune characteristics of the prognostic risk model based on ERSRGs in osteosarcoma

**Fig. 6:**
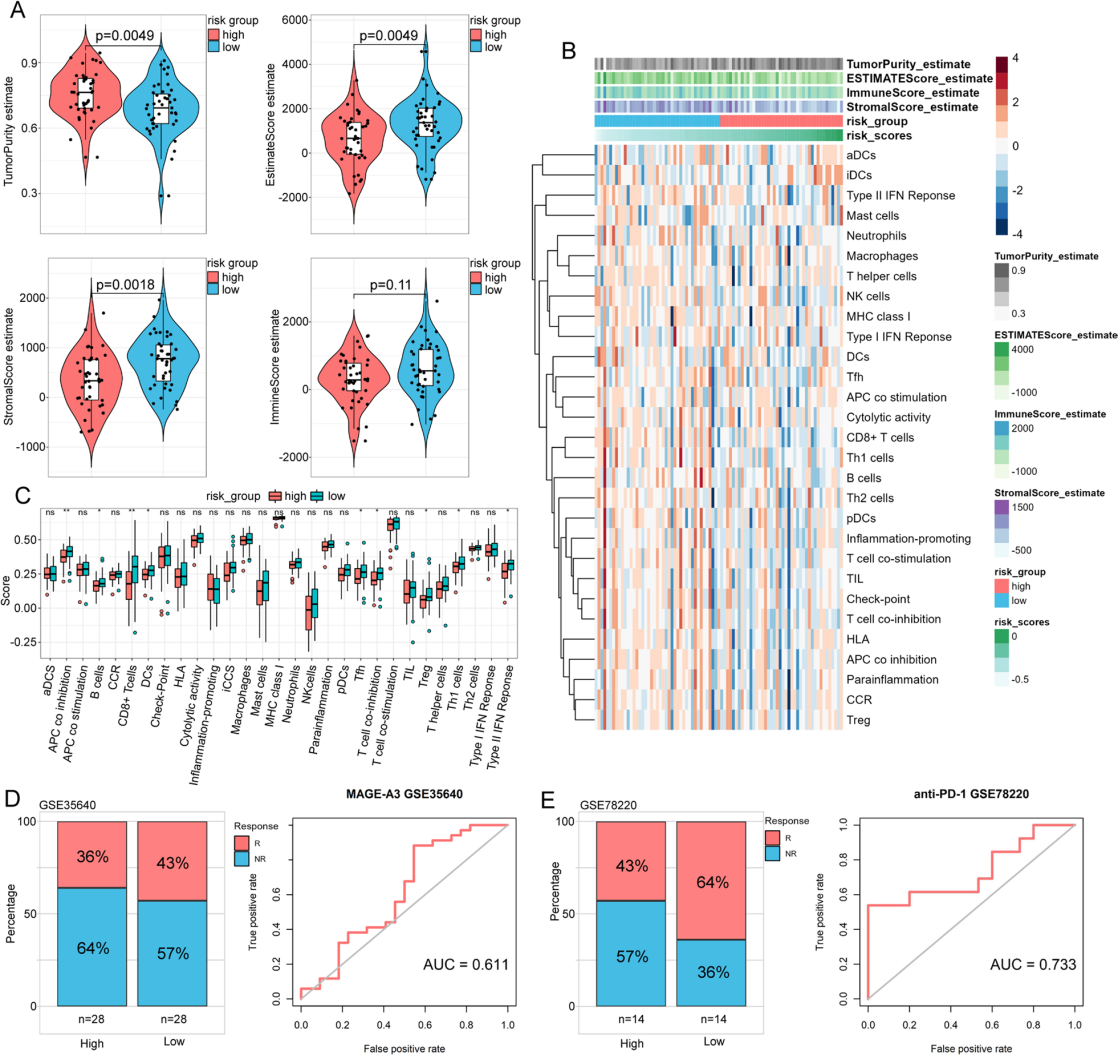
Correlation of the ERSRPS with the immune landscape in osteosarcoma patients. **A** Violin plot showing the differences in the immune score, stromal score, estimate score, and tumor purity between the low- and high-risk groups. **B**, **C** Enrichment levels of immune-related cells and pathways between the low- and high-risk groups. **D**, **E** The proportion of patients responded to immunotherapy and ROC analysis between the low- and high-risk groups in the GSE35640 cohort and GSE78220 cohort. R, Response; NR, Non-response; *p < 0.05, **p < 0.01, ***p < 0.001

Given the aforementioned findings, ER stress was likely related to the immune microenvironment in osteosarcoma. To gain further insight into the relationship between ER stress and the immune characteristics of osteosarcoma patients, we compared the differences in immune cell components and tumor cells between the low- and high-risk groups. We found that the high-risk group exhibited a lower estimate score (*p* = 0.0049), immune score (*p* = 0.11), and stromal score (*p* = 0.0018) but a higher tumor purity (*p* = 0.0049) than the low-risk group (Fig. 6A). We then utilized the ssGSEA algorithm to calculate the scores for 29 immune-related gene sets encompassing various immune cell types and functions, in each sample (Fig. 6B). This allowed us to characterize the tumor immune microenvironment (TIME) status in the TARGET-OS cohort. The results showed that the low-risk score group had significantly higher levels of tumor-infiltrating immune cells, including B cells, CD8+ T cells, dendritic cells (DCs), T follicular helper (Tfh) cells, regulatory T (Treg) cells, and Th1 cells (Fig. 6C). Moreover, the scores of immune-related pathways, including APC co inhibition, T-cell co-inhibition, and type II interferon (IFN) response, were significantly higher in the low-risk group (Fig. 6C).

Based on the relationship between the risk model and immune components, we further explored the prediction of the score for prognosis and immunotherapy response in two additional external validation sets (GSE35640, GSE78220) containing patient immunotherapy response. In both validation sets, the results demonstrated that the non-response rate was higher among high-risk patients undergoing treatment (Fig. 6D, E). These data provided new insights into the high-risk group’s poorer prognosis, mainly due to the lower infiltration of immune cells, and consequently, a potentially reduced responsiveness to immunotherapy.

### CMap screened and validated the anti-osteosarcoma effect of ISOX in in vitro and vivo

**Fig. 7:**
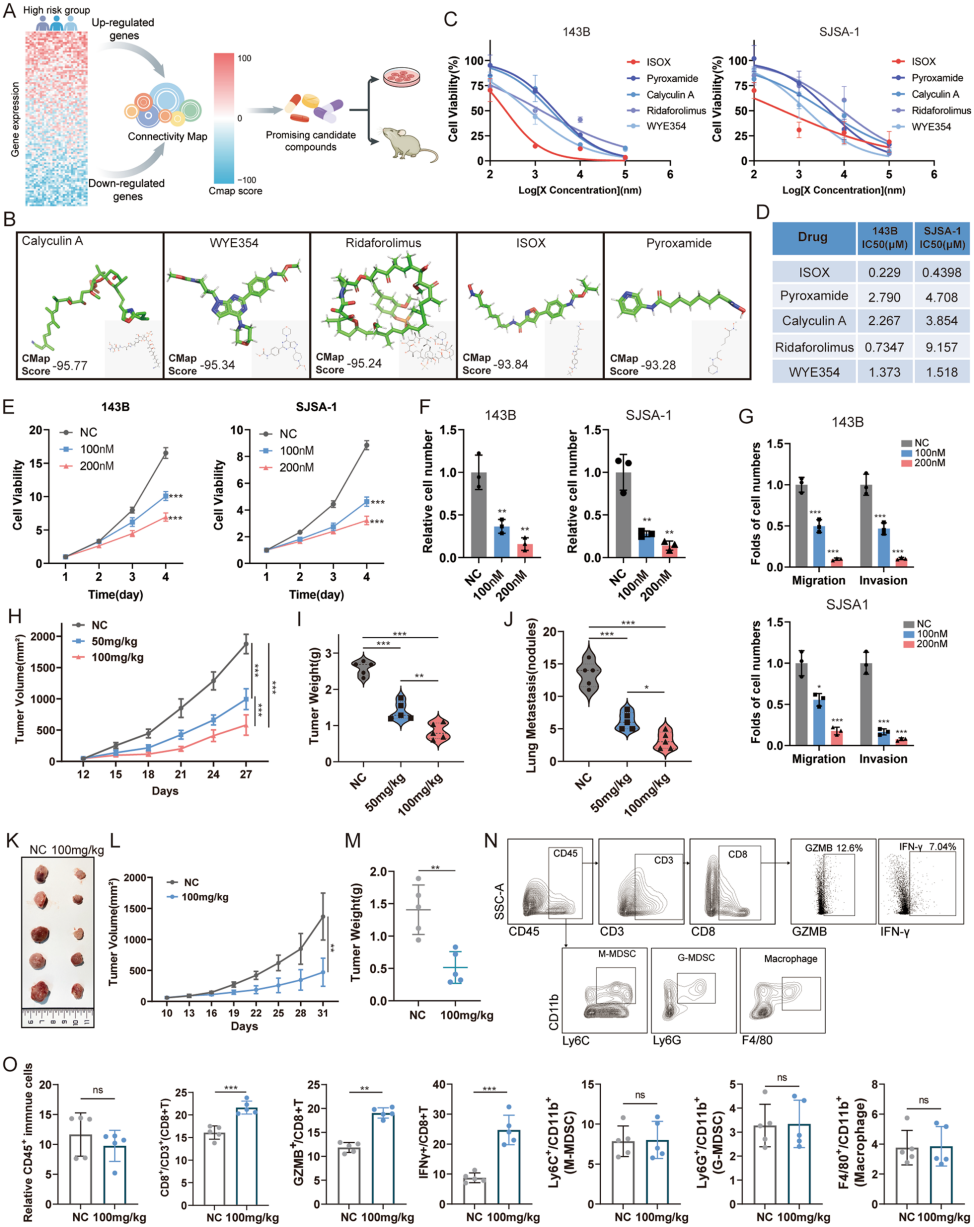
ISOX exerts the anti-osteosarcoma effect. **A** Schematic showing steps for identifying potential anti-osteosarcoma drugs base on CMap database. **B** Molecular structural formulas of small molecular compounds with CMap scores less than − 90. **C**, **D** IC50 of five small molecular compounds in 143B and SJSA-1 cells according to the CCK-8 assay. **E** Cell proliferation, **F** colony formation, **G** migration and invasion abilities were assessed in ISOX-treated 143B and SJSA-1 cells. **H**, **I**, **J** Tumor volume, tumor weight and the number of lung metastasis nodules in BALB/C nude mouse tibial orthotopic models with ISOX 50 mg/kg/daily and 100 mg/kg/daily treatment (n = 5 per group). **K**, **L**, **M** General view, tumor volume, tumor weight in K7M2 syngeneic tumors with ISOX 100 mg/kg/daily treatment (n = 5 per group). **N**, **O** CD8+ T cells, G-MDSC, M-MDSC, macrophage and CD8+ T cell functional markers analysed by flow cytometry. *p < 0.05, **p < 0.01, ***p < 0.001

Considering the lower survival rate observed in the high-risk group, it is imperative to investigate subgroup-specific therapeutic agents for these patients. To identify potential therapeutic agents for patients in the high-risk group, we uploaded 100 upregulated, and 100 down-regulated differentially expressed ERSRGs between the high- and low-risk groups to the CMap database (Fig. 7A). As a result, we were able to identify the top five compounds (Calyculin, Deforolimus, ISOX, WYE-354, and Pyroxamide) with negative scores ≤ − 90 (Fig. 7B), suggesting their potential potent antitumor effects in high-risk group patients. We then conducted in vitro and in vivo experiments to evaluate the efficacy of the five compounds. Initially, the 48 h half-maximal inhibitory concentration (IC50) of the compounds was determined on the proliferation of 143B and SJSA-1 osteosarcoma cells. The results demonstrated that these five small compounds significantly inhibited the survival of osteosarcoma cells (Fig. 7C, D). ISOX consistently exhibited the lowest IC50 in both 143B and SJSA-1 cells (222.9 and 439.8 nM, respectively), indicating its higher potency than the other four compounds (Fig. 7D). Moreover, ISOX dose-dependently inhibited the ability of proliferation (Fig. 7E), colony formation (Fig. 7F, Additional file 4: Figure S2A, B), and suppressed migration and invasion (Fig. 7G, Additional file 4: Figure S2C, D) in 143B and SJSA-1 cells. We further exploited in vivo experiments utilizing orthotopic animal models to assess the impact of ISOX treatment on tumor growth and metastasis. The results showed that ISOX treatment significantly inhibited SJSA-1 tumor growth. This was evident from the observed differences in xenograft tumor size (Fig. 7H, Additional file 4: Figure S2E), weight (Fig. 7I) and lung metastasis (Fig. 7J, Additional file 4: Figure S2F) between the treatment and control group. To further investigate the role of ISOX treatment in regulating TME, we conducted an in *vivo* experiment with syngeneic osteosarcoma cell line K7M2. We found that both tumor volume (Fig. 7K, L) and weight (Fig. 7M) were reduced by ISOX treatment. In flow cytometry analysis, we observed that ISOX treatment induced an increased infiltration of CD8+ T cells within tumors, notably comprising functional IFN-γ+ CD8+ T cells and Granzyme B+ CD8+ T cells (Fig. 7N, O). In addition, there were no significant alterations in the populations of CD45+ immune cells, G-MDSC, M-MDSC, and macrophage (Fig. 7N, O). These consistent results provided compelling evidence for the anti-osteosarcoma effect of ISOX.

## Discussion

Various metabolic and oncogenic abnormalities perturb protein folding homeostasis in malignant and infiltrating immune cells in the tumor microenvironment [4]. The sustained activation of ER stress enables malignant cells to adapt to environmental challenges and coordinates multiple immune regulatory mechanisms to promote malignant progression, metastasis, and therapy resistance. Presently, persistent ER stress has emerged as a novel hallmark of cancers, and interventions targeting ER stress hold promise for destabilizing aggressive cancer cells and augmenting antitumor immunity [23, 24].

To elucidate the specific role of ER stress and its complex interaction with the immune microenvironment in osteosarcoma, we developed and validated a prognostic risk score model based on patents RNA-sequencing data from the TARGET-OS, GSE33382, and GSE21257 cohorts. Five prognostic ERSRGs were then screened and used to construct the ERSRPS. Among the five DE-ERSRGs, the Kaplan–Meier survival analysis suggested that osteosarcoma patients exhibiting high expression levels of BCL2, MAGEA3, MAP3K5, and TXNDC12 had significantly improved overall survival rates. In contrast, patients with high expression levels of STC2 had shorter overall survival time. B-cell lymphoma-2 (Bcl2) is a classical mitochondrial pro-apoptosis regulatory protein that inhibits the intrinsic programmed cell death pathway [25]. BCL2 inhibitors have been reported to elicit only modest responses in specific patient subsets with solid cancers [26]. Melanoma-associated antigen-A3 (MAGEA3), a prominent member of cancer testis antigen, is as a potential immunotherapeutic target due to its elevated expression in various malignant tumor cells [27]. Mitogen-Activated Protein Kinase Kinase Kinase 5 (MAP3K5), a member of the mitogen-activated protein kinase (MAPK) family, plays a vital role in processes such as apoptosis, secretion of inflammatory cytokines and survival [28]. Thioredoxin domain-containing protein 12 (TXNDC12), a member of the protein disulfide isomerase (PDI) family, is implicated in the tumorigenicity of human gastric cancer [29] STC2, also known as Stanniocalcin 2, is a secreted glycoprotein that exhibits significant transcriptional and post-transcriptional regulation under a variety of stress conditions, such as ER stress, hypoxia, and nutrient deprivation. It plays a crucial role in cellular stress response by aiding cells in adapting to adverse environments and preventing apoptosis [30]. Studies have reported its widespread expression in various tumor cells and tissues, including breast, colorectal, stomach, esophageal, prostate, kidney, liver, bone, ovarian, and lung [30, 31]. Overexpression of STC2 has been shown to be associated with increased cell proliferation, migration, and development of acquired resistance to chemotherapy and radiotherapy [30]. In this study, we demonstrated that STC2 expression was upregulated in osteosarcoma tissues, which was correlated with a poorer survival. Targeting STC2 significantly suppressed the growth and metastasis of osteosarcoma cells. These findings suggests that STC2 could be a biomarker for osteosarcoma prognosis and treatment. In addition, STC2 expression was found to be negatively correlated with Type II IFN response and CD8+ T cell infiltration based on TARGET-OS cohort, which were reported to associated with the induction of antitumor immunity and responsiveness to ICB therapy [32, 33]. Previous studies have indicated that cancer cells evade immune surveillance mainly through suppressing the expression of major histocompatibility class I (MHC-I), which, in turn, hinders both the antitumor effect of type II IFN response and the infiltration of CD8+ T cell [34]. Loss of MHC-I expression in tumor cells enables them to evade recognition and destruction by cytotoxic T lymphocytes [35]. Notably, our study revealed a remarkable increase in MHC-I molecules following STC2 knockdown in osteosarcoma cells, implying that STC2 may mediate immune response by downregulating the expression of MHC-I molecules on the surface of tumor cells. Furthermore, as a secreted glycoprotein, the in vitro osteosarcoma cells and CD8+ T cells co-culture system demonstrated that STC2 hampered the antitumor effect of cytotoxic CD8+ T cell by reducing the expression of IFN-γ and Granzyme B. Collectively, we demonstrated that the ER stress related gene STC2 has the potential to modulate immune suppression by reducing MHC-I molecule expression in osteosarcoma cells and impairing CD8+ T cell function within the TME.

The TME consists both tumor cells and various infiltrating immune cells. The intricate composition and complex cell-to-cell interactions play a crucial role in driving tumor growth, metastasis, and modulating the immune responses against cancer [36, 37]. In addition, a body of researches have indicated that cancer or immune cell-intrinsic ER stress responses can impact malignant progression by altering immune cell functionality that coexist in the TME [38–40]. In this study, our research revealed that ER stress related gene STC2 contributed to immune suppression. Thus, exploring the differences within the TME based on the ER stress-related prognostic signature may provide valuable insights into the underlying tumor biology between the groups and lead to development of comprehensive therapeutic strategies. In our study, the low-risk group had higher estimate and stromal scores, implying a more favorable immune microenvironment with increased immune cells infiltration. Immune-related pathways analysis further unveiled that the observed immune differences between the groups were primarily attributable to infiltrating T cells, particularly CD8+ T cells, Th1 cells, and TIL. In contrast, the high-risk group exhibited higher tumor purity, indicating a greater proportion of malignant cells and an immune-cold tumor microenvironment. These characteristics could account for the anticipated diminished responsiveness to immune checkpoint inhibitors (ICBs) in the high-risk group when compared to the low-risk group. Together, our findings implied that excessive activation of ER stress may enhance tumor cell aggressiveness and suppress infiltrating immune cells in osteosarcoma patients in the high-risk group, thereby contributing to a poor survival rate.

Given the aforementioned findings, it is of the utmost importance and urgency to identify potent therapeutic drugs for patients in high-risk group. In recent years, research on existing drugs has revealed their potential to exert therapeutic effects by interacting with and targeting molecules beyond their established pathways or targets [41, 42]. This creates promising opportunities for repurposing existing drugs for new clinical indications, given that these drugs have already undertaken human safety testing, thereby facilitating expedited clinical translation. The CMap database holds substantial importance in elucidating functional connections between small molecule compounds, genes, and disease states [43]. In this study, we utilized the CMap database to screen five potential therapeutic drugs that may benefit patients in high-risk group. We validated the significant inhibitory effects of these drugs on osteosarcoma cell growth through in vitro experiments. Additionally, our research provided compelling evidence, both at the cellular level and in an animal model, to support the most significant inhibitory effects of ISOX on the growth and metastasis of osteosarcoma. ISOX, also known as CAY10603, has been reported as a selective inhibitor of histone deacetylase 6 (HDAC6) [44]. Significantly, HDAC6 is a key regulator involved in the degradation of misfolded proteins through the formation of aggresomes [45]. Hou et al. reported that ISOX held therapeutic potential for diabetic nephropathy by suppressing NLRP3 inflammasome activation in macrophages and tubular cells [46]. In addition, ISOX has recently been considered as a future therapeutic for cholangiocarcinoma [47]. Previous studies have shown that some pharmacological ER stress modulators, including IRE1α, PERK, eIF2α, and BiP inhibitors, exhibit antitumor effects in preclinical cancer models [48–50]. In our study, we verified the anti-osteosarcoma effect of the selective HDAC6 inhibitor ISOX through ER stress-related signature screening. Furthermore, our findings suggested that ISOX treatment may exert anti-osteosarcoma effects by modulating the infiltration of functional CD8+ T cells. This unveils novel insights into the HDAC6 inhibitor ISOX in anti-tumor responses. Further investigations focusing on the drug-molecule interactions are warranted to provide more conclusive evidence.

Several limitations of our study should be acknowledged. Firstly, the ERSRPS was developed and validated retrospectively using publicly available databases. Additionally, the currently accessible osteosarcoma databases encompass relatively modest sample sizes. Therefore, prospective research is imperative to assess the clinical applicability of the signature through extensive multi-center trials for future clinical implementation. Furthermore, comprehensive functional experiments and investigations into drug-molecule interactions are necessary to uncover the intricate mechanisms underlying ISOX’s anti-osteosarcoma effects.

## Conclusions

In this study, patients were divided into high- and low-risk subgroups based on five prognostic ERSRGs. The constructed ER stress-related signature exhibited a robust predictive performance for overall survival and immune status. Additionally, we identified that ER stress related gene STC2 promoted the malignant development and metastasis in osteosarcoma, and modulated immune responses by reducing MHC-I molecule expression in osteosarcoma cells and impairing CD8+ T cell function within the TME. Furthermore, ISOX was found to be an effective anti-osteosarcoma small molecule drug that holds promise for repurposing in osteosarcoma.

### Supplementary Information

**Additional file 1: Table S1.** Summary of 62 osteosarcoma patients clinical information.

**Additional file 2: Table S2.** Immunohistochemistry-related indexes for the 62 patients

**Additional file 3: Figure S1.** Analysis of STC2 in pan-cancer. (A) Expression of STC2 in different types of cancers. (B) Correlation between STC2 expression and immune cell infiltration in different cancer types. The correlation between STC2 expression and TMB (C) as well as MSI (D) across 33 cancer types. (E) Co-expression analysis of STC2 and immune checkpoints in pan-cancer. *p < 0.05, **p < 0.01, ***p < 0.001.

**Additional file 4: Figure S2.** ISOX exerts the anti-osteosarcoma effect. (A, B) ISOX treatment inhibited colony formation, (C, D) migration and invasion abilities in 143B and SJSA-1 cells. (E, F) ISOX treatment inhibited tumor growth and lung metastasis in BALB/C nude mouse.

## Data Availability

Publicly available datasets were analyzed in this study. These data can be found in: https://www.ncbi.nlm.nih.gov/geo/query/acc.cgiacc=GSE21257,GSE33382,GSE35640,GSE78220 and https://ocg.cancer.gov/programs/target.

## Abbreviations

STC2: Stanniocalcin 2
ER: Endoplasmic reticulum
ERSRGs: Endoplasmic reticulum stress-related genes
ERSRPS: Endoplasmic reticulum stress-related prognostic signature
UPR: Unfolded protein response
MHC-I: Major histocompatibility class I
ICBs: Immune checkpoint blockades
TIME: Tumor immune microenvironment
TCGA: The Cancer Genome Atlas
GEO: Gene Expression Omnibus
LASSO: Least absolute shrinkage and selection operator
OCLR: One-class logistic regression
IHC: Immunohistochemistry
GZMB: Granzyme B
