# Supplementary figures and images for: Construction of an ER stress-related prognostic signature for predicting prognosis and screening the effective anti-tumor drug in osteosarcoma
Source: J Transl Med. 2024 Jan 16;22:66. doi: 10.1186/s12967-023-04794-0 (PMC10792867; doi:10.1186/s12967-023-04794-0)

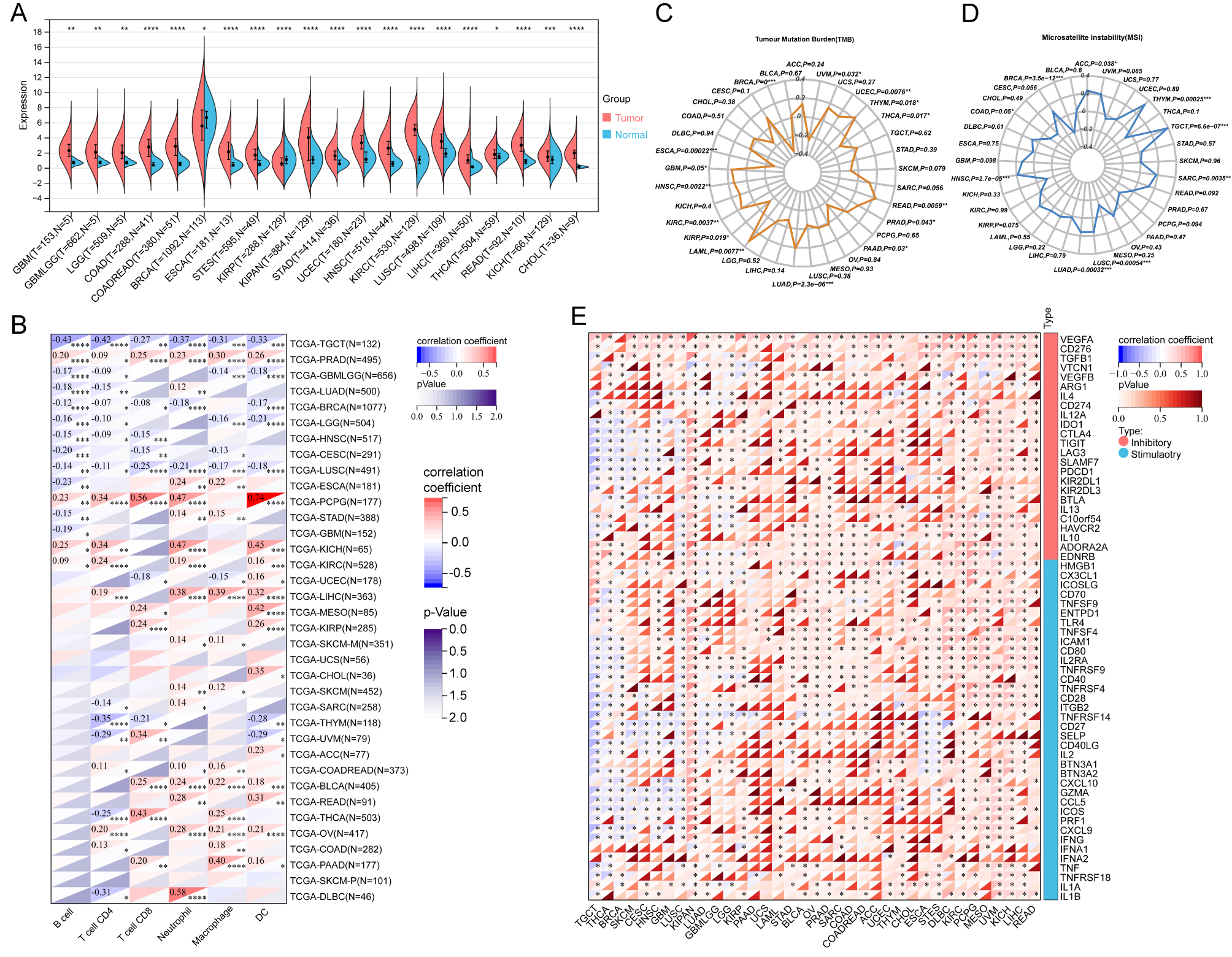

Supplement: Supplementary file 3 — Additional file 3: Figure S1. Analysis of STC2 in pan-cancer. (A) Expression of STC2 in different types of cancers. (B) Correlation between STC2 expression and immune cell infiltration in different cancer types. The correlation between STC2 expression and TMB (C) as well as MSI (D) across 33 cancer types. (E) Co-expression analysis of STC2 and immune checkpoints in pan-cancer. *p < 0.05, **p < 0.01, ***p < 0.001. [file 12967_2023_4794_MOESM3_ESM.tif]

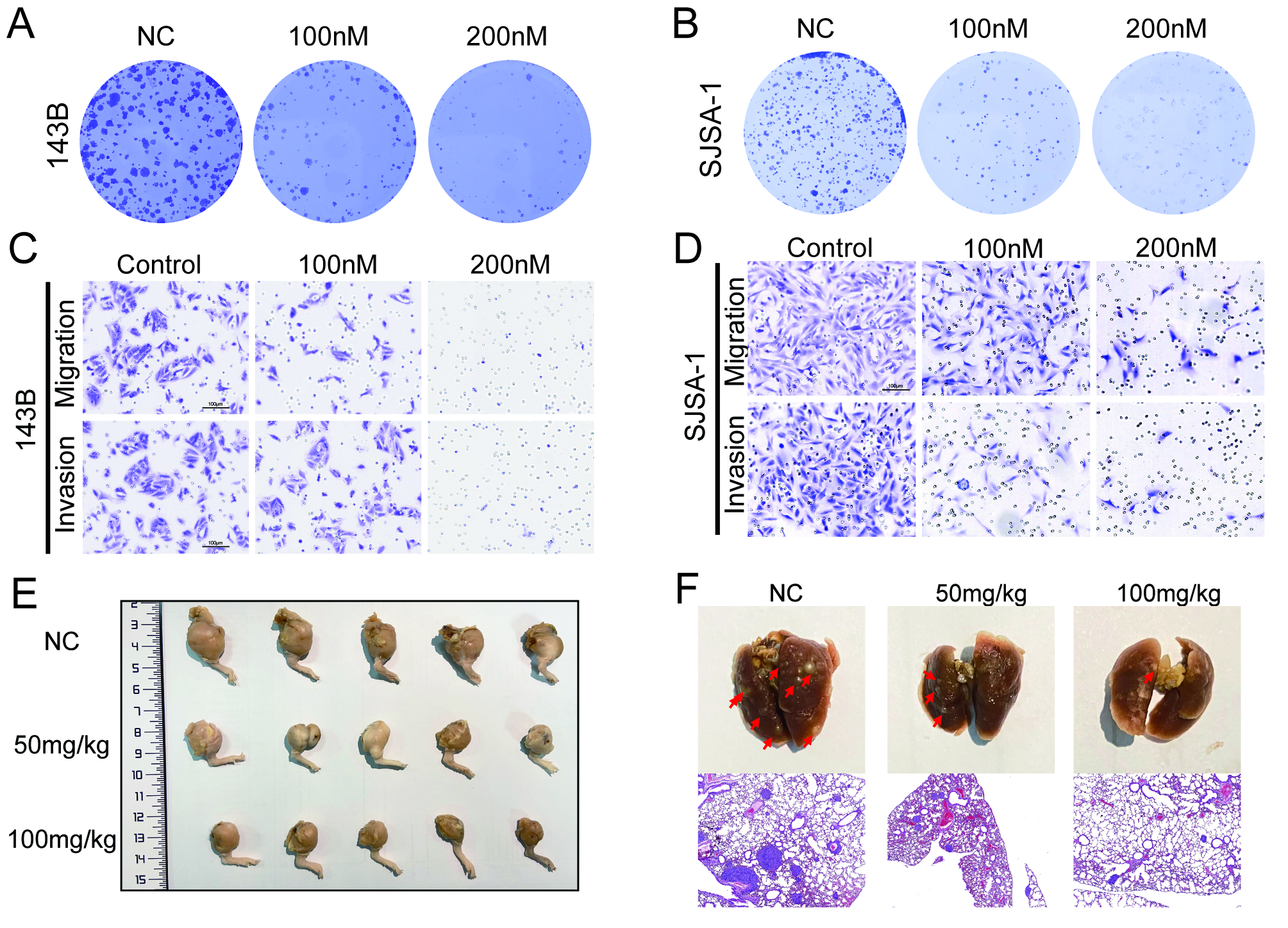

Supplement: Supplementary file 4 — Additional file 4: Figure S2. ISOX exerts the anti-osteosarcoma effect. (A, B) ISOX treatment inhibited colony formation, (C, D) migration and invasion abilities in 143B and SJSA-1 cells. (E, F) ISOX treatment inhibited tumor growth and lung metastasis in BALB/C nude mouse. [file 12967_2023_4794_MOESM4_ESM.tif]
